# Arginine GlcNAcylation and Activity Regulation of PhoP by a Type III Secretion System Effector in *Salmonella*

**DOI:** 10.3389/fmicb.2021.825743

**Published:** 2022-01-20

**Authors:** Juan Xue, Yuxuan Huang, Hua Zhang, Jiaqingzi Hu, Xing Pan, Ting Peng, Jun Lv, Kun Meng, Shan Li

**Affiliations:** ^1^Institute of Infection and Immunity, Taihe Hospital, Hubei University of Medicine, Shiyan, China; ^2^College of Life Science and Technology, Huazhong Agricultural University, Wuhan, China; ^3^College of Biomedicine and Health, Huazhong Agricultural University, Wuhan, China; ^4^Shanghai Fengxian District Central Hospital, Shanghai, China

**Keywords:** *Salmonella*, SseK3, arginine GlcNAcylation, PhoP, DNA-binding ability

## Abstract

*Salmonella* type III secretion system (T3SS) effector SseK3 is a glycosyltransferase delivered directly into the host cells to modify host protein substrates, thus manipulating host cellular signal transduction. Here, we identify and characterize the Arg-GlcNAcylation activity of SseK3 inside bacterial cells. Combining Arg-GlcNAc protein immunoprecipitation and mass spectrometry, we found that 60 bacterial proteins were GlcNAcylated during *Salmonella* infection, especially the two-component signal transduction system regulatory protein PhoP. Moreover, the Arg-GlcNAcylation of PhoP by SseK3 was detected *in vivo* and *in vitro*, and four arginine residues, Arg65, Arg66, Arg118, and Arg215 were identified as the GlcNAcylation sites. Site-directed mutagenesis showed that the PhoP R215A change significantly reduced the DNA-binding ability and arginine to alanine change at all four sites (PhoP 4RA) completely eliminated the DNA-binding ability, suggesting that Arg215 is essential for the DNA-binding activity of PhoP and GlcNAcylation of PhoP affects this activity. Additionally, GlcNAcylation of PhoP negatively regulated the activity of PhoP and decreased the expression of its downstream genes. Overall, our work provides an example of the intra-bacterial activities of the T3SS effectors and increases our understanding of endogenous Arg-GlcNAcylation.

## Introduction

*Salmonella*, a Gram-negative bacterium, can cause a broad range of food-borne diseases, including gastroenteritis, enteric fever, and bacteremia in a large range of mammalian hosts (Bell et al., 2005). *Salmonella enterica* serovar Typhimurium (*S*. Typhimurium) encodes two specific type III secretion systems (T3SSs) within *Salmonella* pathogenicity islands 1 and 2 (SPI-1 and SPI-2) that display functions during infection (Hansen-Wester and Hensel, 2001). The effectors of the SPI-1 T3SS can cause a ruffle of the intestinal epithelial cell membrane to engulf the bacteria, which resembles the process of phagocytosis (Takaya et al., 2003). After *Salmonella* infecting the host cells, the bacterial cells remain within a modified phagosome called the *Salmonella*-containing vacuole (SCV), in which they will survive and replicate with nutrient limitations (Dandekar et al., 2014). However, *S*. Typhimurium has evolved a large amount of regulatory circuits that facilitate them to adapt to the nutrient-limited environment. One important regulatory system is the PhoQ/PhoP two-component signal transduction system, which is essential for *Salmonella* virulence (Groisman, 2001).

Arginine glycosylation is a newly identified post-translational modification. It is unusual because it glycosylates a poor nucleophile arginine (Li et al., 2013; Pearson et al., 2013). A conserved family of SPI-2 effectors named SseK in *Salmonella* and NleB in both *Escherichia coli* and *Citrobacter rodentium* have been reported. These effectors show novel arginine glycosyltransferase activity that modifies the target proteins by attaching *N*-acetyl glucosamine (GlcNAc) on specific arginine residues (Li et al., 2013; Pearson et al., 2013; Luo et al., 2015; Park et al., 2018; Ding et al., 2019; Araujo-Garrido et al., 2020; Pan et al., 2020). Crystal structure study reveals that NleB, SseK1, SseK3 belong to the GT-A family glycosyltransferase (Esposito et al., 2018; Park et al., 2018; Ding et al., 2019; Araujo-Garrido et al., 2020; Pan et al., 2020). Multiple host protein substrates for the NleB/SseK orthologs have been described. TRADD, FADD, RIPK1, HIF-1α, and GAPDH are reported to be the host targets for EPEC NleB (Gao et al., 2013; Li et al., 2013; El Qaidi et al., 2017; Gunster et al., 2017; Scott et al., 2017; Ding et al., 2019; Newson et al., 2019; Xue et al., 2020a; Garcia-Garcia et al., 2021). We and other groups have reported TRADD is a *in vivo* substrate of NleB and SseK1. Arg-GlcNAcylation on TRADD by NleB and SseK1 is crucial for bacterial pathogenesis (Newson et al., 2019; Xue et al., 2020a). SseK3 was also found to affect bacterial virulence by modifying signaling receptors TNFR1, TRAILR, and phylogenetically related Rab GTPases (Newson et al., 2019; Gan et al., 2020; Meng et al., 2020; Xue et al., 2020a). In addition to glycosylating host proteins, NleB, SseK1, and SseK3 could also glycosylate themselves (Park et al., 2018; Newson et al., 2019; Xue et al., 2020b; Garcia-Garcia et al., 2021). However, most of these researches have focused on proteins related to the host immune response.

Multiple other studies have highlighted the Arg-GlcNAcylation of bacterial proteins, and the functional of such glycosylation are still unclear (Scott et al., 2017; Newson et al., 2019). Two recent studies have revealed that intra-bacterial Arg-glycosylation contributes to bacterial survival (El Qaidi et al., 2020, 2021). Yet, there is little systematic research on Arg-GlcNAcylated proteins within *S.* Typhimurium. Therefore, in this study we used mass spectrometry to identify a series of intra-bacterial proteins with Arg-GlcNAcylation. The results showed that *Salmonella* SseK extensively glycosylated proteins during *Salmonella* infection. Particularly, SseK3 glycosylates the two-component regulatory system PhoP on Arg65, Arg66, Arg118, and Arg215. Mutation of Arg215 abolished the DNA-binding activity of PhoP, and GlcNAcylation of PhoP affected this activity. Besides, GlcNAcylation of PhoP affects the expression of its downstream-regulated genes. Therefore, SseK3 glycosylates a two-component regulatory system protein PhoP, thus inhibiting the activity of PhoP with a negative feedback regulation. These findings also provide new perspectives for understanding the post-translational modification of PhoP.

## Materials and Methods

### Bacterial Strains and Plasmids Construction

Bacterial strains and plasmids used in this study were listed in Supplementary Table 1. Wild-type *phoP*, as well as its derivatives, were cloned into a modified pTrc99a vector (in which the C-terminal multiple cloning sites were replaced with 1x Flag sequence) and pET28a vector. For complementation in *S.* Typhimurium Δ*sseK1/2/3*, *sseK1*, *sseK2*, and *sseK3*, together with their upstream promoter regions, were inserted into the pET28a vector as described previously (Meng et al., 2020; Xue et al., 2020b). Furthermore, *sseK3* was also inserted into the pGEX-6P-2 vector for protein expression in *E. coli*. All point mutations were generated by overlap PCR. All plasmids were verified by Sanger DNA sequencing.

### Bacterial Growth Assays

The overnight bacterial culture of *Salmonella* was diluted by 1:20 in 50 mL LB medium or LPM medium with pH 5.8 as described previously (Coombes et al., 2004). Cultures were grown at 37°C, shaking at 220 rpm with the following antibiotics: kanamycin (50 μg/mL) (1758-9316, INALCO), ampicillin (100 μg/mL) (17589314, INALCO), streptomycin (50 μg/mL) (1758-9319, INALCO), and monitored by an automated plate reader.

### Antibodies and Reagents

The anti-GlcNAc arginine antibody (ab195033, Abcam) was described previously (Pan et al., 2014). Antibodies for Flag M2 (F2426) and DnaK 8E2/2 (ab69617) were purchased from Sigma and Abcam, respectively. Unless otherwise mentioned, the cell culture products were purchased from Invitrogen, and all other reagents were Sigma-Aldrich products.

### Cell Culture

293T cells obtained from the American Type Culture Collection (ATCC) were grown in Dulbecco’s Modified Eagle’s Medium (DMEM) (HyClone) supplemented with 10% fetal bovine serum (FBS), 2 mM L-glutamine, 100 U/mL penicillin, and 100 μg/mL streptomycin. For the infection experiment, the media does not contain any antibiotics. Cells were cultivated in a humidified atmosphere containing 5% CO_2_ at 37°C.

### Infection Experiment and Isolation of Intracellular Bacteria

The method was performed as described previously (Meng et al., 2020; Xue et al., 2020b). Briefly, 293T cells were seeded to 10 cm dishes at a concentration of 5 × 10^6^ cells per dish 1 day before infection. WT and mutant *Salmonella* strains were inoculated into 10 mL LB medium containing appropriate antibiotic and incubated overnight (∼12 h). The bacterial culture was then subcultured (1:33) in LB without antibiotics for another 3 h. Bacterial inoculates were diluted in serum-free and antibiotics-free DMEM, and infection was performed at a multiplicity of infection (MOI) of 20 at 37°C, 5% CO_2_ for 30 min. At the endpoint of infection, extracellular bacteria were removed by extensive washing with pre-warmed phosphate-buffered saline (PBS), and culture media was replaced with media containing 100 μg/mL gentamicin for a further 1.5 h. Culture media was finally replaced with media containing 20 μg/mL gentamicin for 12–14 h.

The isolation of intracellular bacteria was conducted according to the protocol of Liu et al. (2012). After the infection, cells were lysed in a buffer containing 0.5% Triton X-100, 20 mM Tris–HCl (pH 7.6) and 150 mM NaCl. The sample was first pelleted at 300 *g* for 5 min to remove host cell nuclei, and then the post-nuclear supernatant was centrifuged again at 3,000 *g* for 20 min. The resulting bacterial pellets were washed extensively with RIPA buffer to minimize host-protein contamination.

### Recombinant Protein Expression

Protein expression was induced overnight in *S.* Typhimurium SL1344 strain at 22°C or in *E. coli* BL21 (DE3) at 16°C with 0.4 mM Isopropyl β -D-1-Thiogalactopyranoside (IPTG) after OD_600_ reached 0.8–1.0. The cells were collected by centrifugation at 4,000 *g* for 20 min and lysed by sonication. The lysates were centrifuged twice at 12,000 rpm at 4°C for 30 min to remove insoluble cell fragments. According to the corresponding methods, the supernatants of Flag tag proteins, 6x His tag proteins, and GST tag proteins were purified with Flag M2 beads, Ni-NTA agarose (GE Healthcare), and glutathione sepharose (GE Healthcare), respectively (Li et al., 2013). For GlcNAcylated-PhoP, His-PhoP was co-expressed with GST-SseK3 in *E. coli* BL21 (DE3) and purified with Ni-NTA agarose (GE Healthcare) as mentioned above. The protein concentration was examined by SDS-PAGE with BSA standards, followed by Coomassie Blue staining.

### Immunoprecipitation

The immunoprecipitation assay of Flag M2 beads and Protein A/G plus Agarose beads (Santa Cruz, CA, United States) was performed according to the manufacturer’s instruction. Briefly, aliquots of 20 μL of Flag M2 beads were washed twice with 0.2 mL immunoprecipitation buffer (50 mM Tris–HCl, 150 mM NaCl, pH 7.6), then mixed with the remaining soluble protein fraction (∼10 mg/mL) and incubated at 4°C. After 4-h incubation, beads were washed three times with Buffer C (50 mM Tris–HCl, 150 mM NaCl, 0.5% Triton X-100, pH 7.6). Bound protein was eluted by 1x SDS sample buffer at 95°C for 20 min, followed by standard immunoblotting analysis. All the immunoprecipitation assays were performed more than three times, and representative results were shown in the figures.

For Arginine-GlcNAcylated proteins enrichment, bacteria were lysed by sonication in immunoprecipitation buffer, supplemented with a protease inhibitor mixture (Roche Molecular Biochemicals). The supernatants were mixed with anti-Arg-GlcNAc antibody beads and incubated at 4°C for 8 h. The beads were washed three times with Buffer C and the immunoprecipitates were eluted by SDS sample buffer at 95°C for 20 min. Input and eluted samples were electrophoresed by standard immunoblotting analysis.

### Gene Expression Data Collection and Analysis

Strains information and RNA-seq expression data were collected from Gene Expression Omnibus (GEO) database^1^ ^,2^. Using fastp to trim reads to remove low-quality reads and adapter sequences, and using STAR (2.7.0c) to map to *Salmonella* reference genome (accession numbers: NC_016857.1 and NC_016810.1) (Dobin et al., 2013; Chen et al., 2018). FeatureCounts (v1.6) was used to calculate the reads numbers mapped to each gene (Yang et al., 2014). Transcripts per million (TPM) was used to draw a heat map.

### Mass Spectrometry Analysis of Tryptic Peptide

To determine the exact Arg-GlcNAcylation site of *Salmonella* endogenous proteins, the immunoprecipitates were separated by SDS-PAGE and subjected to in-gel trypsin digestion. The final peptide samples were analyzed by the Q Exactive Plus mass spectrometer equipped with nanoflow reversed-phase liquid chromatography (EASY nLC 1200, Thermo Scientific). EASY nLC 1200 was fitted with a Thermo Scientific Acclaim PepMap nano-trap column (C18, 5 μm, 100 Å, 100 μm × 2 cm), a Thermo Scientific EASY-Spray column (PepMap RSLC, C18, 2 μm, 100 Å, 50 μm × 15 cm), and run at 300 nL/min with the following mobile phases (A: 0.1% formic acid; B: 80% acetonitrile/0.1% formic acid). The liquid chromatography separation was carried out with the following gradient: 0∼3% B for 4 min, 3∼28% B for 30 min, 28∼80% B for 2 min, 80% B for 4 min, 80∼100% B for 5 min. Eluted peptides were electro sprayed directly into the mass spectrometer for MS and MS/MS analysis in a data-dependent acquisition mode. One full MS scan (350–1500 m/z) was acquired, then immediately the 10 ions with the highest intensity were selected for MS/MS analysis by high-energy collisional dissociation (HCD) fragmentation. Dynamic exclusion was set with a repeat duration of 24 s and exclusion duration of 12 s. In-source collision-induced dissociation (CID) was set to 0 and normalized collision energy (NCE) was set to 27%.

### Mass Spectrometry Data Analysis

Mass spectrometry proteomics raw data were collected from PRIDE (identifier PXD010769), integrated proteome resources (iProX) (identifier IPX0001304001), and our lab. Identification of proteins and Arg-glycosylated peptides was accomplished using Proteome Discoverer™ 2.2 with HT-Sequest. Searches were performed against *Salmonella* Typhimurium SL1344 (UniProt proteome id UP000008963) proteomes database with carbamidomethylation of cysteine set as a fixed modification and the variable modifications of oxidation of methionine, HexNAc (Arg-GlcNAc, Ser-GlcNAc, Thr-GlcNAc, Asn-GlcNAc), and acetylation of protein N-termini. The precursor mass tolerance was set to 10 ppm with a False Discovery Rate (FDR) of 1% for protein and peptide filter. KEGG and Gene Ontology (GO) enrichment analysis were annotated by KAAS^3^, and UniProt^4^ and plots were performed using clusterProfiler in R (Yu et al., 2012).

### *In vitro* GlcNAcylation Assay

*In vitro* GlcNAcylation assay was performed as described previously (Meng et al., 2020). Forty microliters reaction mixtures, including 5 μg PhoP protein or its mutants, 1 μg GST-SseK3, 1 mM UDP-GlcNAc, 2 mM MnCl_2_, 20 mM HEPES pH 7.5, and 150 mM NaCl were incubated at 37°C for 2 h. Reactions were terminated by boiling at 95°C for 5 min in SDS-PAGE sample buffer, followed by 10% SDS-PAGE and subjected to western blot analysis.

### Electrophoretic Mobility Shift Assay

The electrophoretic mobility shift assays (EMSAs) was performed as described previously (Su et al., 2021). Briefly, EMSAs were performed using the purified PhoP or its variants and DNA probe 6′FAM labeled *phoP* promoter. DNA probe was amplified from *Salmonella* Typhimurium SL1334 genomic. The probe (1 nM) was mixed with various amounts of proteins in 20 mL of EMSA binding buffer (25 mM Tris–HCl, 50 mM KCl, 5 mM MgCl_2_, 0.5 mM EDTA, and 10% glycerol, pH 8.0). After incubation at room temperature for 30 min, the samples were analyzed by 5% non-denaturing polyacrylamide gel electrophoresis at 4°C. The gels were photographed by using a gel imaging system (Fujifilm FLA7000). The assay was repeated at least three times, and a representative result was shown.

### qRT-PCR

qRT-PCR was performed with the SYBR Green I RNA-to-CT2-step kit (Vazyme) according to the manufacturer’s instructions and a CFX96 Touch real-time PCR detection system (Bio-Rad). The *gyrB* mRNA or 16s rRNA was served as *Salmonella* reference transcripts. Fold changes in expression were determined using the 2^–ΔΔCt^ method (Ye et al., 2011). Primer sequences are given in Supplementary Table 2, and their specificities have been confirmed using Primer-BLAST (NCBI).

### Structure Homology Models

The server SWISS-MODEL was used to create structural homology models (Template PDB ID: 4s04). All structure files were evaluated by PROBITY^5^. All structure figures were prepared in PyMOL (Schrödinger, LLC) and Discovery Studio 2019.

### Statistical Analysis

All the results are presented as mean ± SD from at least three independent experiments. Statistical analysis was performed using two-tailed Student’s *t*-test and indicated as follows: *P < 0.05, ^**^P < 0.01, ^***^P < 0.001, n.s., not significant. The KEGG and GO enrichment analysis were employed by Hypergeometric statistical test, and the Benjamini and Hochberg FDR multiple testing correction.

### In-Gel Trypsin Digestion

Samples were separated by SDS-PAGE, visualized with Coomassie G-250 or by ProteoSilverä PlusSilver Stain Kit (SIGMA, United States) according to protocol instructions. Bands were excised and destained in 50% acetonitrile (ACN) and 50 mM NH_4_HCO_3_ solution with shaking at room temperature. Destained samples were washed with the buffer containing 300 μL 100% ACN, and the buffer was removed after 10 min incubation, following by vacuum-drying for at least 10 min. Dehydrated samples were then incubated with the reducing buffer containing 300 μL DTT solution (10 mM DTT in 50 mM NH_4_HCO_3_) at 56°C. For 1-h incubation, the reducing buffer was removed and the reduced samples were washed with 100% ACN to remove residual DTT, following by vacuum-drying for at least 10 min. Reduced samples were subsequently alkylated with 60 mM Iodoacetamide (IAM) in 50 mM NH_4_HCO_3_ in the dark for 30 min at room temperature. Alkylated samples were hydrated with 100% ACN for 10 min incubation. Reduced and alkylated samples were then digested with enough trypsin for 16 h at 37°C. Trypsin was removed and the digested peptides were collected by extraction buffer (100% ACN, 0.1% FA). Peptides were desalted using C18 stage tips and dried by concentrator at 60°C and analyzed by a Q Exactive Plus mass spectrometer.

## Results

### Activation of *Salmonella* SPI-2 Genes *sseK1*/2/3 in LPM Media Increased the Level of Bacterial Endogenous Arg-GlcNAcylation

**FIGURE 1:**
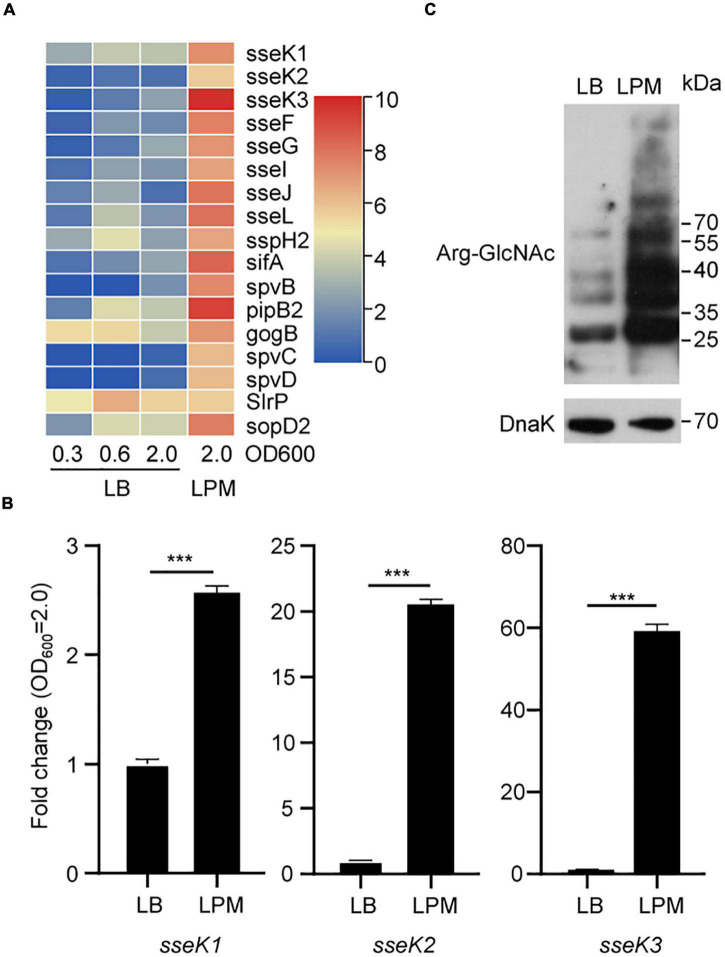
A bunch of endogenous proteins within *Salmonella* could be arginine-GlcNAcylated in the LPM medium. **(A)** Heat map analysis of *Salmonella* transcriptomes under different culture conditions in the GEO database. **(B)** Detection of *sseK1/2/3* in *Salmonella* lysates by qRT-PCR. The indicated *Salmonella* strains were grown in LB or LPM to an OD600 of 2.0 and total RNA was extracted and served as the template for qRT-PCR measurements. The stably expressed *gyrB* mRNA was used as an internal reference. Values are means (bars) ± standard deviations (SD) (error bars) from six biological replicate experiments. ***P < 0.001. **(C)** Western blot analysis of the Arg-GlcNAcylation level of the whole cell lysates. The wild type *Salmonella* strain SL1344 was grown in LB or in LPM medium to an OD600 of 2.0 (early stationary phase). Bacteria were harvested and samples were prepared, followed by standard immunoblotting analysis with the indicated antibodies. Data in **(B,C)** are from at least three independent experiments.

*Salmonella* SPI-2 genes play a central role in bacterial replication and survival in macrophages. Acidic, low-phosphate, low-magnesium medium (LPM) can simulate the growth conditions of *Salmonella* in SCV (Kroger et al., 2013; Westermann et al., 2019). To evaluate the expression of *Salmonella* SPI-2 genes in LPM, we reanalyzed *Salmonella* transcriptomes in the Gene Expression Omnibus (GEO) public repository. We found that SPI-2 genes were indeed upregulated under LPM condition, indicating that low Mg^2+^ and acidic pH can activate SPI-2 genes including *sseK1/2/3* gene family (Figure 1A and Supplementary Table 3). Furthermore, unlike SPI-2 genes *spvB*, *spcC*, and *spcD*, *sseK1/2/3* were expressed in the early exponential phase and stationary phase of LB medium according to the RNA-seq results (Supplementary Table 3). Therefore, for further detecting the expression of *sseK1/2/3* in *Salmonella*, the designated *Salmonella* SL1334 strain was grown in LB or LPM media to an OD_600_ of 2.0, and the total RNA was extracted and served as the template for qRT-PCR measurements. Comparing with culturing in LB medium, *sseK1/2/3* gene expression were upregulated in LPM medium. Especially for *sseK3*, it was significantly activated in LPM medium with about 80-fold changes (Figure 1B). Subsequently, we analyzed the GlcNAcylation of the whole cell lysates by western blot. The results showed that activation of *sseK1/2/3* increased the levels of Arg-GlcNAcylation and caused more endogenous Arg-GlcNAcylation of *Salmonella* (Figure 1C). Taken together, these results suggested that LPM media induced *Salmonella* SPI-2 genes expression, especially *sseK1/2/3*, thus increasing the levels of bacterial endogenous Arg-GlcNAcylation.

### Mass Spectrometry Analysis Determined the Two-Component Regulatory System Protein PhoP Is a GlcNAcylated Protein During *Salmonella* Infection

**FIGURE 2:**
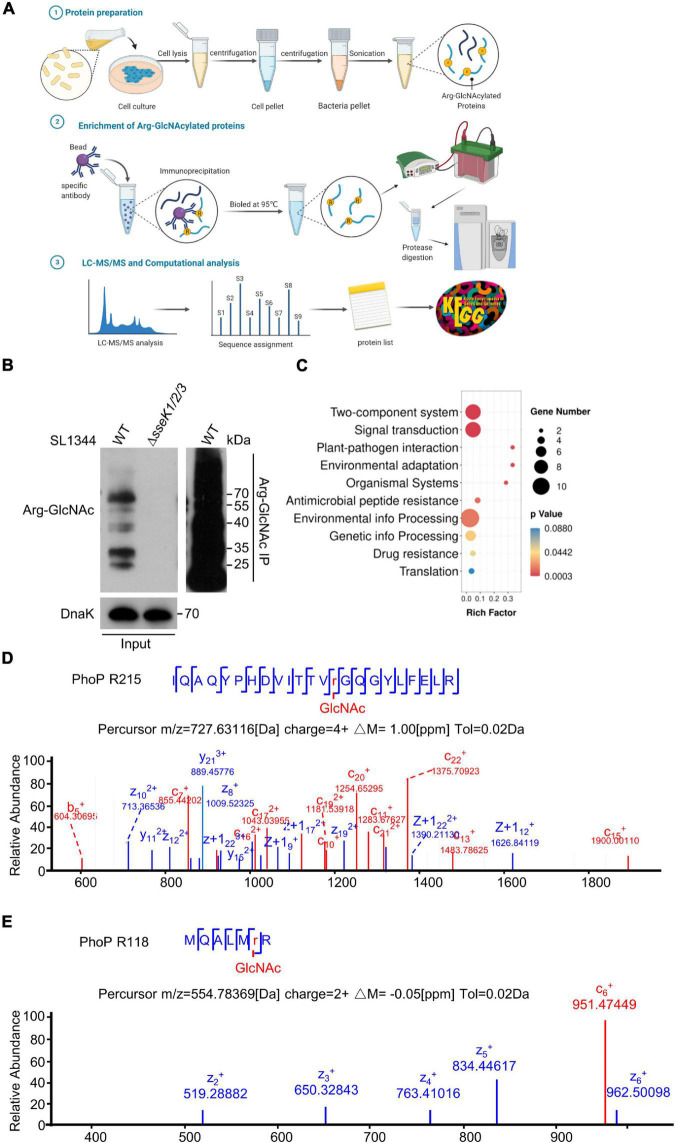
Mass spectrometry analysis determined the two-component regulatory system protein PhoP was a GlcNAcylated protein during *Salmonella* infection. **(A)** Schematic diagram of the preparation of bacterial proteins under simulated infection conditions, Arg-GlcNAcylated proteins enrichment, and computation analysis of Arg-GlcNAcylated peptides. The infected 293T cells were lysed by 0.5% Triton X-100 and intracellular bacteria were separated by gradient centrifugation. Bacterial proteins were obtained after sonication for subsequent enrichment of glycosylated modified proteins. Protein A/G beads were mixed with anti-Arg-GlcNAc antibody for 6–8 h, followed by incubating the bacterial protein for 8 h. All the proteins were eluted by SDS sample buffer, separated on SDS-PAGE, and digested with trypsin into peptides for further LC-MS/MS analysis. All the Tandem Mass Spectrometry raw data combined with public *Salmonella* proteomic database were analyzed by Proteome Discoverer and annotated *via* KEGG and Gene Ontology. This figure was created with biorender.com. **(B)** Anti-Arg-GlcNAc immunoprecipitations of the intracellular WT *Salmonella* lysate from the infected 293T cells. Bacterial proteins were subjected to anti-Arg-GlcNAc immunoprecipitation and immunoblotting with the corresponding antibodies. Anti-DnaK detection was used as a loading control. **(C)** KEGG analysis of Arg-GlcNAcylated proteins detected by mass spectrometry. The top 10 enrichment pathways are shown in the rainbow bubble chart. The Rich factor is the ratio of the number of Arg-GlcNAcylated protein annotated in this pathway term to all gene numbers annotated in this pathway term. The size of the circle represents the number of modified proteins and the color represents different *P*-values. **(D,E)** Mass spectrum of the PhoP Arg215 and Arg118 peptides. The fragmentation patterns of the generated ions were exhibited along the peptide sequence on top of the spectrum. Data in **(B–E)** are from at least three independent experiments.

To identify the bacterial endogenous arginine GlcNAcylated proteins *in vivo*, we used a modified protocol to isolate enough intracellular bacteria from 293T cells during the infection and perform Arg-GlcNAcylated proteins enrichment for further LC-MS/MS analysis (Liu et al., 2012; Figure 2A). Arg-GlcNAcylation was observed in all biological replicates of wild type *Salmonella* infection, whereas no Arg-GlcNAcylation was detected in negative control Δ*sseK1/2/3* deletion mutant strain (Figure 2B). And our western blot result showed a strong Arg-GlcNAcylation in immunoprecipitation pellet from the intracellular bacteria (Figure 2B). In the combination analysis of *S.* Typhimurium SL1344 proteome database, more than 60 Arg-GlcNAcylated proteins had been identified (Supplementary Figure 1 and Supplementary Table 4). Then we performed GO and KEGG enrichment analysis to assign functional categories to the modified proteins, and found that about 30% of identified proteins are related to the two-component system pathway and signal transduction pathway, indicating that Arg-GlcNAcylation might play a significant role in bacterial signal transduction (Figure 2C and Supplementary Tables 5, 6). Interestingly, upon tandem MS (MS/MS) analysis of Newson’s study and our results, two peptides of two-component regulatory system protein PhoP protein had a mass shift of 203.079 Da (Newson et al., 2019). By MS/MS analysis, the modification sites were mapped to Arg118 and Arg215, respectively (Figures 2D,E). These findings suggested that PhoP was a GlcNAcylated protein.

### SseK3 GlcNAcylated PhoP on Arg65/66/118/215

**FIGURE 3:**
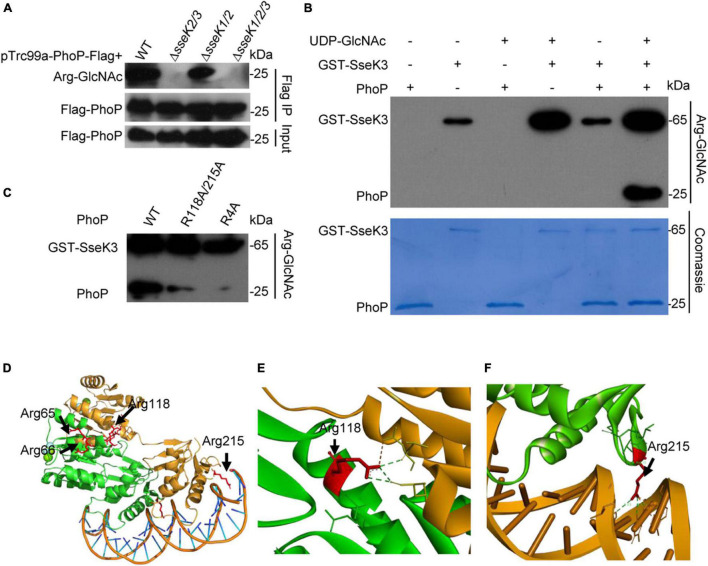
SseK3 modified the two-component regulatory system protein PhoP. **(A)** *In vivo* modification of PhoP. Indicated *Salmonella* strains were grown in the LPM media. Bacteria lysates were collected and were subjected to SDS-PAGE, followed by immunoblotting analyses as shown. **(B)** *In vitro* Arg-GlcNAcylation of PhoP by SseK3. PhoP and GST-SseK3 were purified from *E. coli*. **(C)** *In vitro* Arg-GlcNAcylation of PhoP and site-directed mutants by SseK3. **(D–F)** Simulation of the structure of *Salmonella* PhoP. The GlcNAcylation arginine residues are shown. The structure of *Salmonella* PhoP was built *via* SWISS-MODEL (template Protein Data Bank ID code 4s04) and refined in PyMOL. Data in **(A–C)** are from at least three independent experiments.

Considering that SseK1 and SseK3 are the arginine-specific glycosyltransferases, we tested whether SseK1 or SseK3 glycosylated PhoP or both. Thus, we electrotransformed the pTrc99a-PhoP vector containing the C-terminal Flag tag in the corresponding wild-type *Salmonella* or lacking each possible combination of SseK1, SseK2, and SseK3 to generate the indicated derivative strains. We affinity-purified Flag-tagged PhoP during *Salmonella* growth in the LPM medium. Immunoblotting showed that only strains expressed SseK3 could modify PhoP, while no GlcNAcylation was detected in the Δ*sseK2/3* and Δ*sseK1/2/3* mutants, suggesting that SseK3 catalyzed the GlcNAcylation of PhoP (Figure 3A). And *in vitro* GlcNAcylation assay demonstrated this result, which was consistent with previous finding (Newson et al., 2019; Figure 3B and Supplementary Figure 2A). However, the single mutation of Arg118 and Arg215 or double mutations of these two sites in PhoP cannot completely eliminate the modification of PhoP by SseK3, suggesting that SseK3-mediated GlcNAcylation of PhoP occurred on additional arginine residues (Figure 3C and Supplementary Figure 2B). To determine the GlcNAcylation sites, the recombinant PhoP protein on the background of Arg118/215Ala mutation was co-expressed with SseK3 in *E. coli* BL21 (DE3) strain. Purified proteins were analyzed on the mass spectrometer. The result indicated that Arg65 and Arg66 are the other two GlcNAcylation modification sites (Supplementary Figure 3). Then we replaced the arginine residues by alanine residues to generate PhoP (4RA) (PhoP_R65/66/118/215A_) and the GlcNAcylation activity was measured with an anti-Arg-GlcNAc antibody in the western blot assay. The results showed that the PhoP (4RA) mutants completely abolished the arginine-GlcNAcylation signal, indicating that the Arg65/66/118/215 in PhoP were the *bona fide* modification sites (Figure 3C). Multiple sequence alignment of PhoP in several bacteria, including *S. enterica, E. coli*, *Shigella flexneri*, *C. rodentium*, *Yersinia pestis*, and *Pseudomonas aeruginosa*, revealed that Arg65, Arg118, and Arg215 are all conserved (Supplementary Figure 4). The modification sites were then shown in structures (Figure 3D). Although the predicted structure shows that Arg118 was located within the dimerization region, PhoP Arg118Ala mutant protein displayed the same elution profile as the wild type PhoP protein in size exclusion chromatography, indicating that Arg118 might not be a key amino acid residue for dimerization (Figure 3E and Supplementary Figure 5). Arg215 was located in the DNA-binding motif, which might regulate the PhoP transcription activity (Figure 3F). Therefore, modification of these residues might influence PhoP structure and consequently, regulate the activity of PhoP.

### GlcNAcylation of PhoP Affected Its DNA-Binding Activity

**FIGURE 4:**
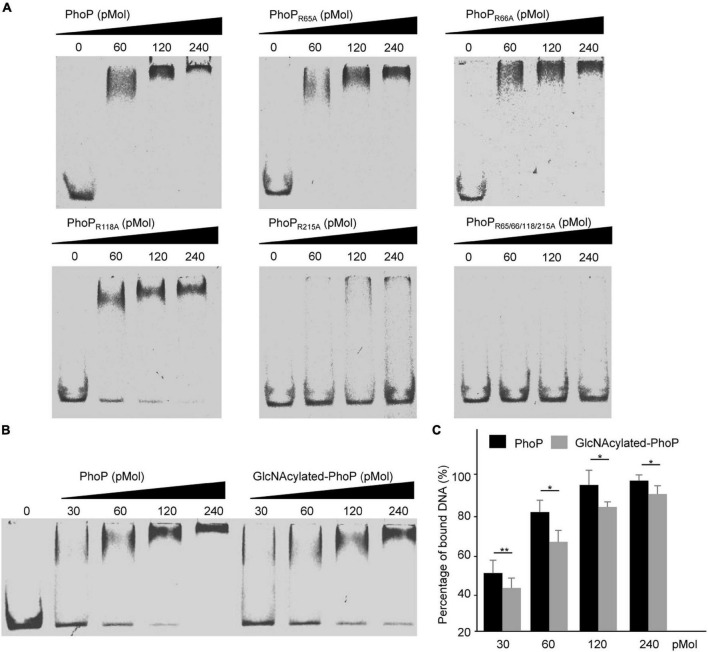
PhoP GlcNAcylation affected its DNA-binding activity. **(A)** DNA binding ability of PhoP variants by EMSA. The indicated amounts of PhoP and its mutants were incubated with PhoP promoter, and then the samples were analyzed by EMSA. **(B)** DNA-binding ability of GlcNAcylated-PhoP. GlcNAcylated-PhoP was purified from His-PhoP co-expressed with pGEX-6P-2-SseK3 in *E. coli* BL21 (DE3). **(C)** Statistical analysis of **(B)**. The percentage of bound DNA was calculated by comparing unbound DNA of 30, 60, 120, and 240 pMol with 0 pMol, respectively. *P < 0.05, **P < 0.01. Data in **(A–C)** are from at least three independent experiments.

PhoP-mediated transcription relies on its binding to PhoP boxes within the promoters of target genes (Lejona et al., 2003; Minagawa et al., 2003). Considering that PhoP can be modified by SseK3, we speculated that GlcNAcylation of PhoP may affect its DNA-binding activity. First, we determined the effects of the above-mentioned four amino acids on PhoP DNA-binding ability. The electrophoretic mobility shift assay (EMSA) was conducted by incubating these proteins with the *phoP* promoter. Results showed that PhoP_R65A_, PhoP_R66A_, and PhoP_R118A_ had a similar DNA-binding activity with wild-type PhoP, while the PhoP_R215A_ significantly reduced the DNA-binding ability. Meanwhile, the PhoP 4RA completely eliminate the DNA-binding ability, suggesting that Arg215 is essential for the DNA-binding activity of PhoP, which was consistent with the location (Figure 4A). Subsequently, we purified GlcNAcylated-PhoP in bacteria. First, we quantified the amount of glycosylation of the modified peptide, which showed Arg215 had the highest modification ratio whether the samples being digested by trypsin (52.1%) or by Glu-C/Lys-C (7.5%) (Supplementary Figures 6, 7). Then we applied it for DNA-binding assays. The results revealed that, comparing with non-GlcNAcylated-PhoP, the GlcNAcylated-PhoP weakened the DNA-binding activity, indicating that GlcNAcylation of PhoP might affect its activity by modifying Arg215 site (Figures 4B,C).

### GlcNAcylation of PhoP Decreased the Expression of Its Downstream Genes

**FIGURE 5:**
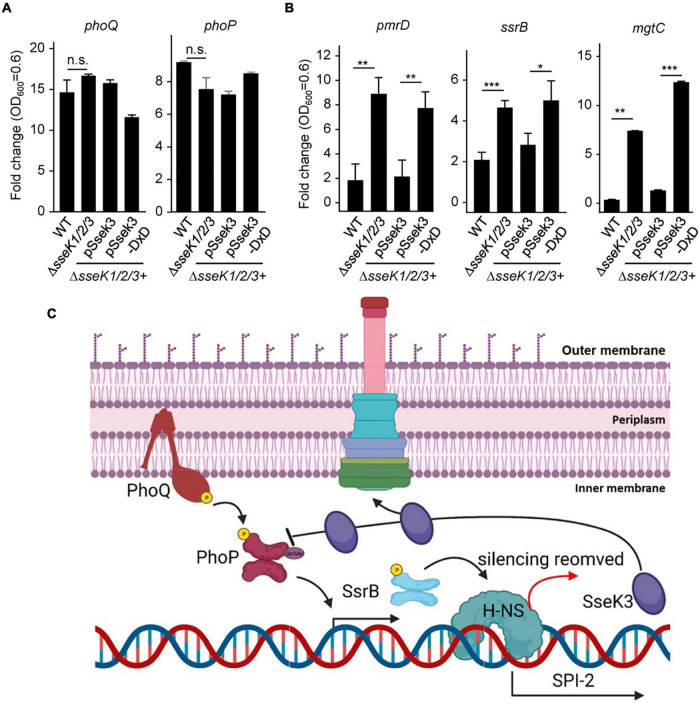
GlcNAcylation of PhoP decreased the expression of its downstream genes. **(A,B)** Expression levels of *phoP*, *phoQ*, *pmrD*, *ssrB*, and *mgtC* in LPM medium. The indicated *Salmonella* strains were grown in the LB and LPM medium to an OD_600_ at 0.6. Total RNA was extracted and served as the template for qRT-PCR measurements. The stably expressed *gyrB* mRNA was used as an internal reference. Values are means (bars) ± standard deviations (SD) (error bars) from six biological replicate experiments. *P < 0.05, **P < 0.01, ***P < 0.001, ns, not statistically significant. **(C)** The working model of SseK3-mediated PhoP GlcNAcylation. The autophosphorylation of kinase sensor PhoQ leads to the phosphorylation of PhoP. PhoP then binds to its target gene promoters, stimulating gene transcription, including SPI-2 genes activator *ssrB*. Activated *ssrB* can relieve the transcriptional inhibition of H-NS and activate a series of SPI-2 genes, including *Salmonella* T3SS effector SseK3. SseK3 is a glycosyltransferase that catalyzes a GlcNAcylation modification of PhoP, thus reducing the binding ability of PhoP to DNA, thereby regulating the expression of SPI-2 genes.

So far, we have demonstrated the importance of Arg-GlcNAcylation in regulating the DNA-binding activity of PhoP, then we want to investigate whether the GlcNAcylation of PhoP is physiologically relevant. We quantified and detected the transcription level of *phoQ/phoP* and downstream-regulated genes, including *pmrD*, *mgtC*, and *ssrB* during the exponential phase. Four corresponding *Salmonella* strains and mutants were used. The wild-type strain (WT) and the complementary strain (pSseK3) can express SseK3. The complementary strain (pSseK3-DxD) is an enzymatic inactive mutant. While the Δ*sseK1/2/3* deletion mutant strain does not express SseK3. The qRT-PCR results showed that the two-component regulatory system gene *phoQ* and *phoP* displayed no obvious difference among these four strains, indicating that GlcNAcylation in *Salmonella* does not promote the expression level of *phoP* at early stage of bacterial growth (Figure 5A). However, the downstream genes *pmrD*, *mgtC*, and *ssrB* were significantly decreased in the strains expressing SseK3 (WT and pSseK3) (Figure 5B). This result suggested that GlcNAcylation of PhoP decreased the expression of its downstream-regulated genes.

## Discussion

SseK3 is an arginine glycosyltransferase that modifies several host proteins such as the signaling receptors TNFR1 and TRAILR to inhibit TNF-stimulated NF-κB signaling and cell death, as well as the small GTPases Rab1 to disrupt ER-to-Golgi trafficking (Newson et al., 2019; Meng et al., 2020; Xue et al., 2020b). One recent transcriptomic research reported that some SPI-2 genes could express during *Salmonella* growth in LB medium and displayed potential intra-bacterial activities, indicating that in addition to target host proteins, SPI-2 genes could also regulate bacteria themselves (Kroger et al., 2013; Westermann et al., 2019; El Qaidi et al., 2020, 2021). In this study, using the 293T cell infection model, we proved the endogenous Arg-GlcNAcylation activity of SseK3 in *Salmonella* and provided an example of the intra-bacterial activities of the T3SS effectors. Newson et al. (2019) previously noted that the Arg-GlcNAcylation of bacterial proteins was occurring during LB growth and infection of RAW264.7 cells, which further corroborated our results. Our data showed that SseK3 glycosylates two-component regulatory system PhoP on Arg65, Arg66, Arg118, and Arg215. Such GlcNAcylation affected PhoP DNA-binding activity and decreased the expression of downstream-regulated genes, with a negative feedback regulation.

Post-translational modifications play an important role in regulating enzyme activities and modulating many biological functions (Deribe et al., 2010; Sang et al., 2016; Ren et al., 2017; Wang et al., 2020). For PhoP-PhoQ, a prominent two-component regulatory system, its importance to bacterial virulence has been reported in almost all bacterial systems (Mitrophanov and Groisman, 2008). Phosphorylation of PhoP is one of the best characterized post-translational modifications, which promotes conformational modifications in its C-terminal DNA-binding domain, and activates *phoP*, thus controlling the expression of 9% *Salmonella* genes, including SPI-2 genes activator *ssrB* (Fass and Groisman, 2009; Colgan et al., 2016; Desai et al., 2016; Choi and Groisman, 2017). Activated *ssrB* can relieve the transcriptional inhibition of H-NS, thereby activating a series of SPI-2 genes including SseK3. In contrast, SseK3 can GlcNAcylate PhoP and reduce the binding ability of PhoP to DNA, thereby regulating the expression of SPI-2 genes (Figure 5C). Arg-GlcNAcylation of PhoP identified in *Salmonella* affected *phoP* DNA-binding activity and negatively regulate the expression of downstream genes, which provides a new type post-translational modification of PhoP and enhances our understanding of PhoP function. In addition to being modified by phosphorylation and Arg-GlcNAcylation, PhoP also processes other post-translational modifications, such as acetylation and methylation, which impairs the PhoP DNA-binding ability, thereby attenuating the virulence of *S*. Typhimurium (Ren et al., 2016, 2017, 2019; Su et al., 2021). Interestingly, we also observed the Arg-GlcNAcylation of OmpR (Supplementary Table 4). The effect of modified OmpR remains unknown and needs further investigation.

Our work raises one important question that remains to be solved about the glycosylation of PhoP in different pathogens. NleB and its orthologs were observed in EHEC, *C. rodentium*, *S*. Typhimurium, and *S. Enteritidi*s (Jennings et al., 2017). It is intriguing whether EHEC, *C. rodentium*, and *S*. *Enteritidis* have a similar regulatory system with arginine glycosylation of PhoP.

In summary, in this study we used mass spectrometry to identify a series of intra-bacterial proteins with Arg-GlcNAcylation. Arg-GlcNAcylation of two-component regulatory systems PhoP on four arginine residues significantly decreases its DNA-binding ability and negatively regulates the expression of downstream-regulated genes, thus providing an example of the intra-bacterial activities of the T3SS effectors. Meanwhile, the exploration of the enzymatic activity of T3SS effectors within bacteria may become a booming research field.

## Data Availability Statement

The original contributions presented in the study are included in the article/Supplementary Material, further inquiries can be directed to the corresponding author.

## Author Contributions

SL and JX conceived the overall study and assisted in the design of the experiments. JX, YH, and HZ conducted and performed the majority of the experiments, and analyzed the data with the assistance from JH, XP, TP, JL, and KM. SL, YH, and JX wrote the manuscript. All authors read and approved the final version of the manuscript.

## Conflict of Interest

The authors declare that the research was conducted in the absence of any commercial or financial relationships that could be construed as a potential conflict of interest.

## Publisher’s Note

All claims expressed in this article are solely those of the authors and do not necessarily represent those of their affiliated organizations, or those of the publisher, the editors and the reviewers. Any product that may be evaluated in this article, or claim that may be made by its manufacturer, is not guaranteed or endorsed by the publisher.

## Footnotes

1
https://www.ncbi.nlm.nih.gov/geo/query/acc.cgi?acc=GSE49829

2
https://www.ncbi.nlm.nih.gov/geo/query/acc.cgi?acc=GSE117256

3
https://www.genome.jp/tools/kaas

4
https://www.uniprot.org/

5
http://molprobity.biochem.duke.edu/
