# Supplementary material for: Arginine GlcNAcylation and Activity Regulation of PhoP by a Type III Secretion System Effector in Salmonella
Source: Front Microbiol. 2022 Jan 20;12:825743. doi: 10.3389/fmicb.2021.825743 (PMC8811161; doi:10.3389/fmicb.2021.825743)
Supplement: Supplementary file 1 [file Data_Sheet_1.docx]

Supplementary Material


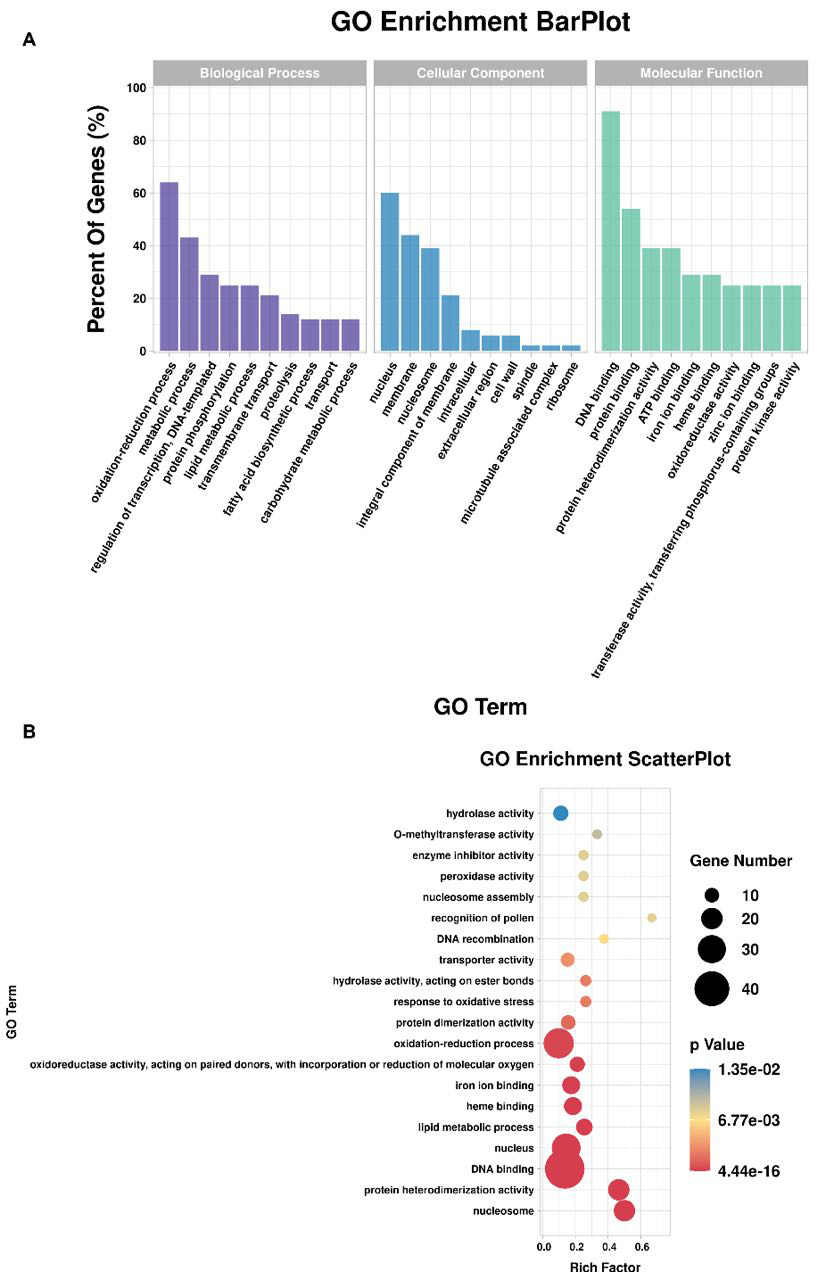


**Figure S1 GO enrichment analysis of Arg-GlcNAcylated proteins. (A)** Bar diagram of functional category enrichment analysis of GlcNAcylated proteins. Y-axis represents the percent of Genes in each category and the X-axis shows the name of the term. **(B)** Scatter plot for GO enrichment results. The top 20 enrichment GO terms are shown in the rainbow bubble chart. The Rich factor is the ratio of Arg-GlcNAcylated protein numbers annotation in this term to all gene numbers annotated in this term. The circle size stands for the number of modified proteins and the color stands for different P-values.


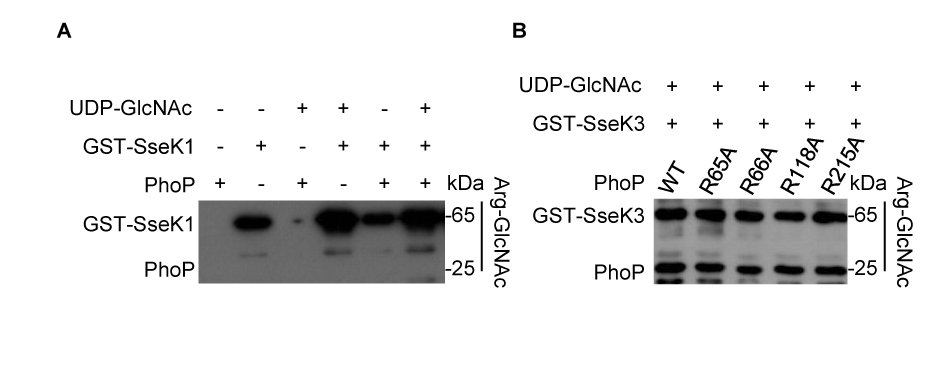


**Figure S2 *In vitro* GlcNAcylation assay of PhoP.** **(A)** Analysis of the enzymatic activity of SseK1 towards the PhoP protein. **(B)** GlcNAcylation activity of single mutations of the modification sites of PhoP in a recombinant reaction *in vitro*. Data in **(A, B)** are representative from at least three repetitions.


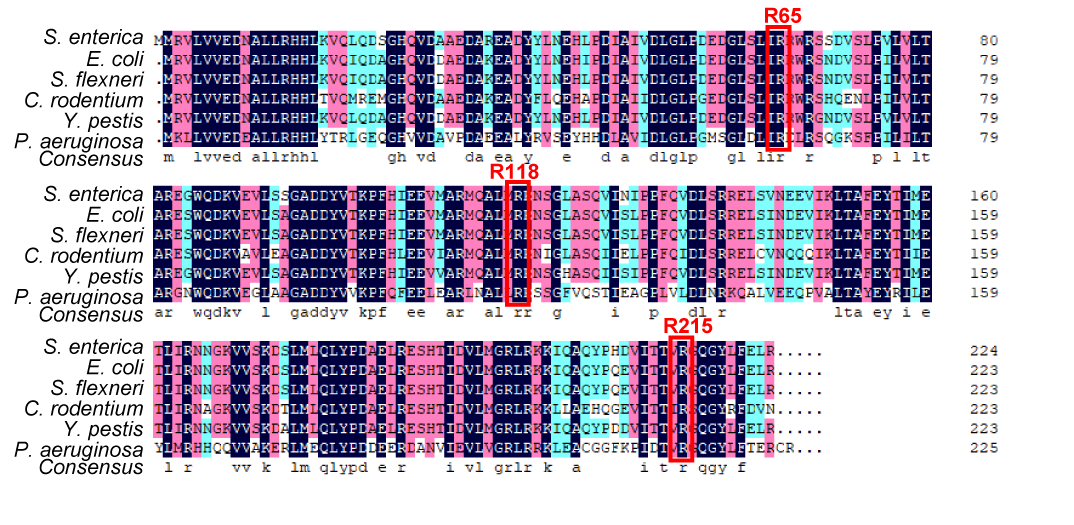


**Figure S3 Multiple sequence alignment of PhoP in several bacteria.** The red box indicates the conservative arginine of PhoP modified by SseK3.


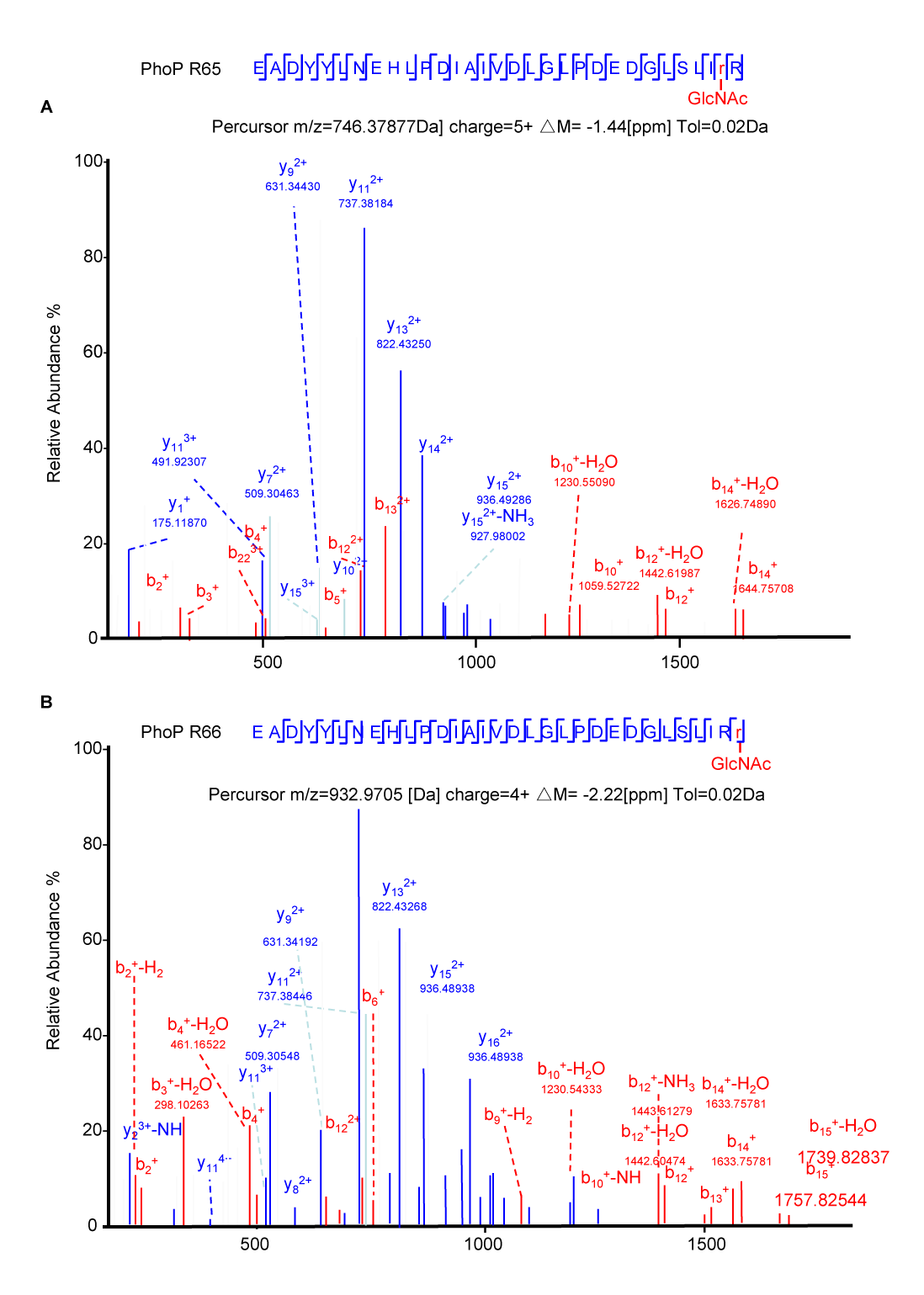


**Figure S4 HCD analysis of the PhoP peptides around Arg65 and Arg66**. HCD mass spectrum of Arg65 (**A**) and Arg66 (**B**) containing tryptic peptides from PhoP catalyzed by SseK3 in bacteria. The fragmentation patterns of the generated ions were exhibited along the peptide sequence on top of the spectrum.


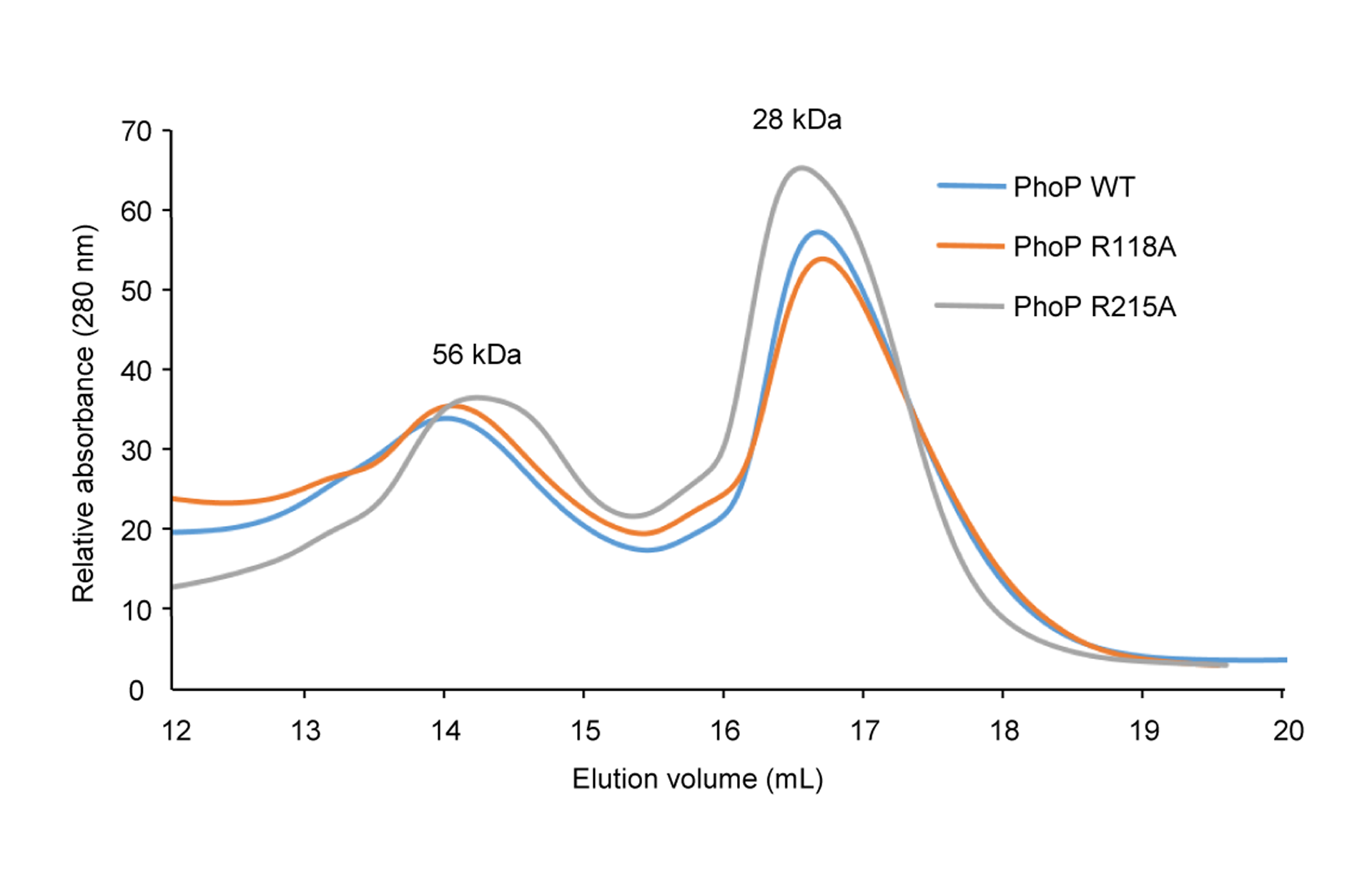


**Figure S5 Size exclusion chromatography of PhoP wild-type protein and PhoP mutants**. Size exclusion chromatography was performed with Superdex 200 increase 10/300GL columns (GE). The column was calibrated with thyroglobulin (669 kDa), ferritin (440 kDa), aldolase (158 kDa), conalbumin (75 kDa), ovalbumin (44 kDa), carbonic anhydrase (29 kDa) and ribonuclease A (13.7 kDa).


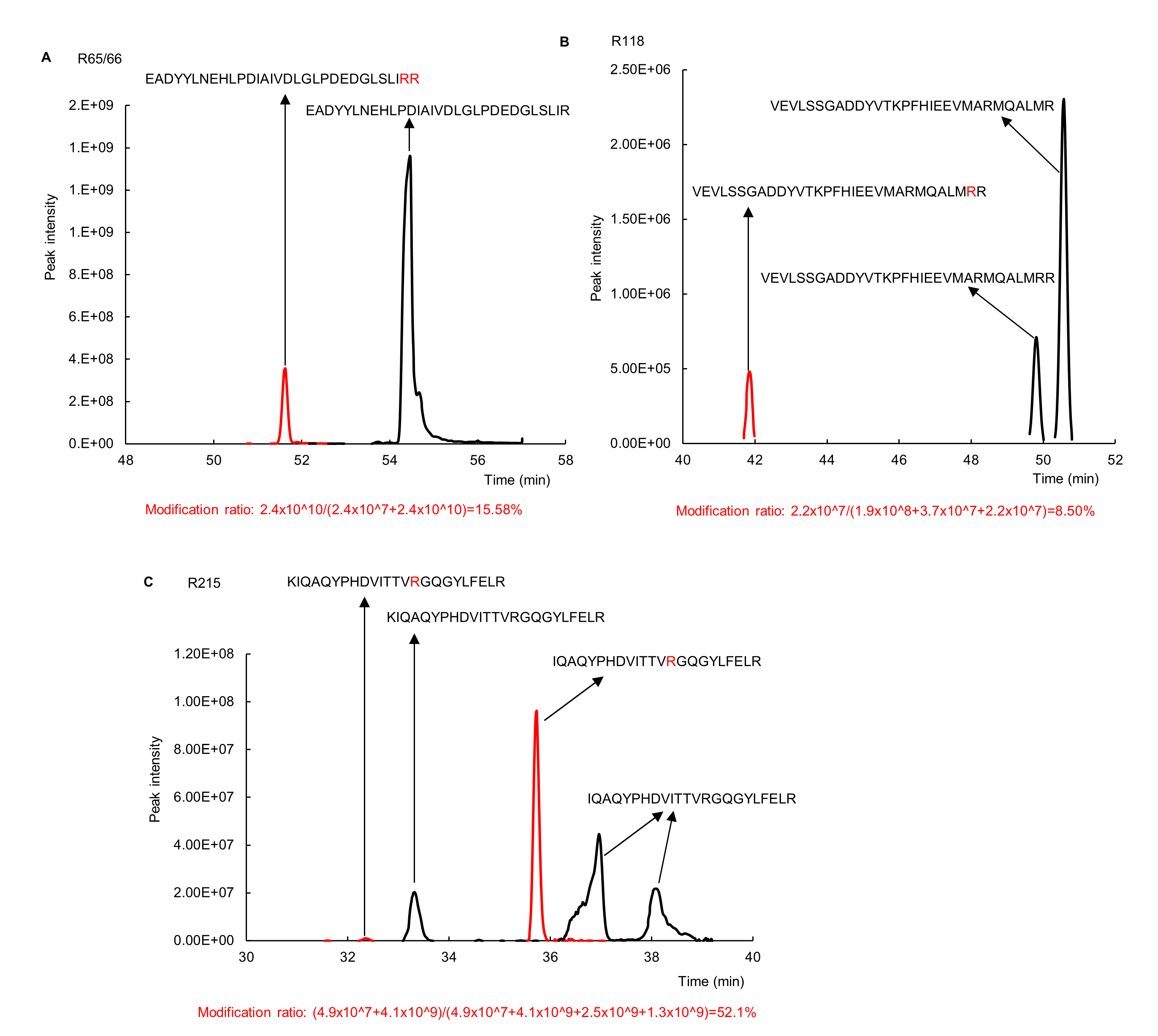


**Figure S6** **Glycosylation ratios of the modified PhoP peptides digested by trypsin.** His-PhoP and GST-SseK3 were co-expressed and purified in *E. coli* BL21 (DE3). The purified His-PhoP was then digested with trypsin and analyzed by LC-MS/MS. Shown are the extracted ion chromatograms of the indicated peptides for R64/65 **(A)**, R118 **(B)**, R215 **(C)**. The peak intensity indicated the relative abundance. The modification ratio was calculated by peak area. The black peak and red peak denote the unmodified and modified peptide, respectively.


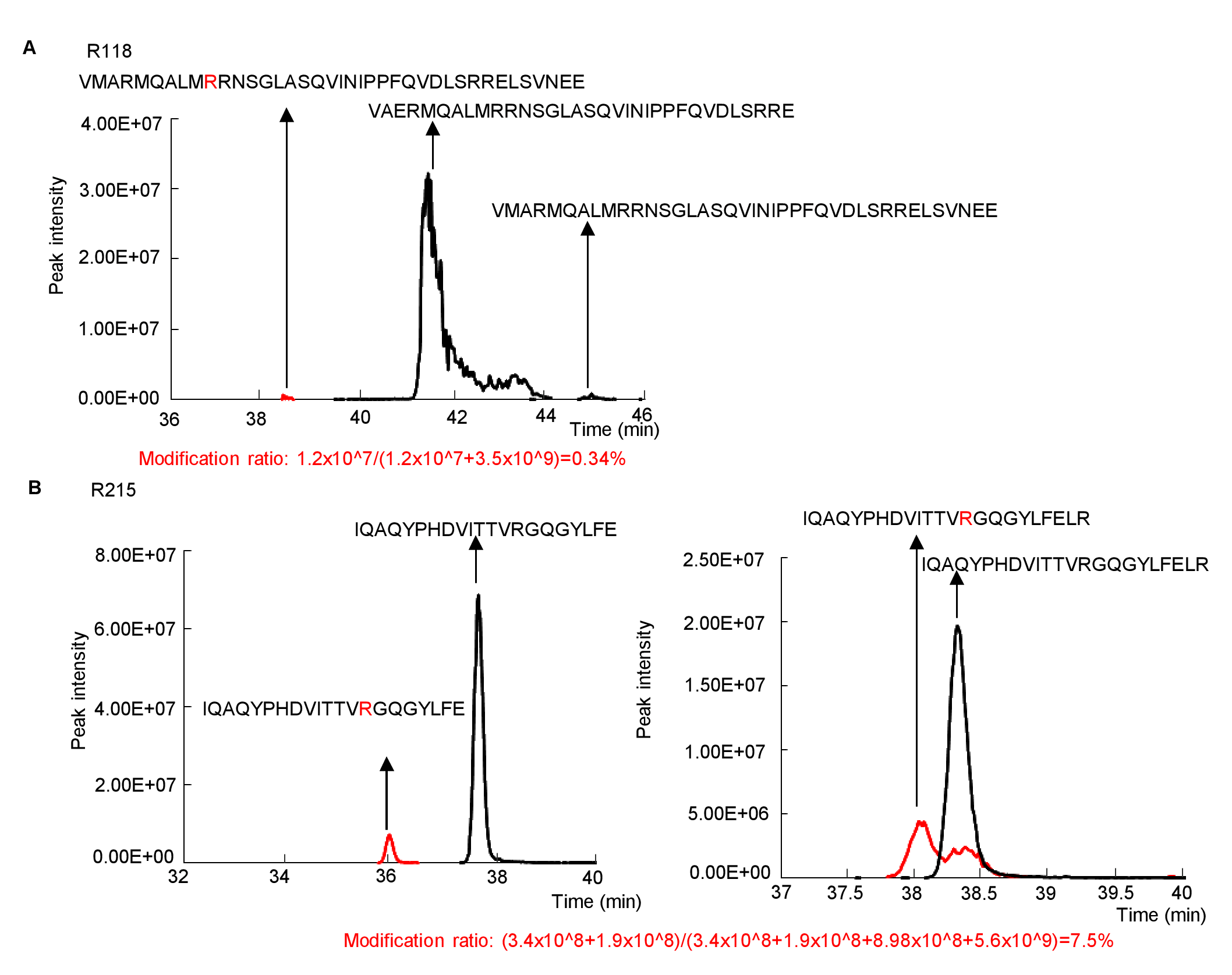


**Figure S7 Glycosylation ratios of the modified PhoP peptides digested by Glu-C/Lys-C.** His-PhoP and GST-SseK3 were co-expressed and purified in *E. coli* BL21 (DE3). The purified His-PhoP was then digested with Glu-C/Lys-C and analyzed by LC-MS/MS. Shown are the extracted ion chromatograms of the indicated peptides for R118 **(A)** and R215 **(B)**. The peak intensity indicated the relative abundance. The modification ratio was calculated by peak area. The black peak and red peak denote the unmodified and modified peptide, respectively.

**Table S1 Strains and plasmids used in this study.**

| **Strains or plasmids** | **Description** | **Sources** |
| --- | --- | --- |
| Strains |  |  |
| *E. coli* TOP10 | *phoP* (Strep^R^) | This study |
| *E. coli* TOP10 | *phop*(R118A) (Strep^R^) | This study |
| *S.* Typhimurium SL1344 | WT(Strep^R^) | Laboratory stock |
| SL1344 ∆123 | *∆sseK1/2/3*(Strep^R^) | (Xue et al., 2020) |
| SL1344 ∆123+pS1 | *∆sseK1/2/3+*pET28a-*sseK1* | (Xue et al., 2020) |
| SL1344 ∆123+pS1(DXD) | *∆sseK1/2/3+*pET28a-*sseK1*(D223A,D225A) | (Xue et al., 2020) |
| SL1344 ∆123+pS3 | *∆sseK1/2/3+*pET28a-*sseK3* | (Xue et al., 2020) |
| SL1344 ∆123+pS3(DXD) | *∆sseK1/2/3+*pET28a-*sseK3*(D226A,D228A) | (Xue et al., 2020) |
| SL1344 ∆123+pPhoP | *∆sseK1/2/3+*pTrc99a-*phoP* | This study |
| SL1344 ∆123+pPhoP(R118A) | *∆sseK1/2/3+*pTrc99a-*phoP*(R118A) | This study |
| SL1344 ∆12+pPhoP | *∆sseK1/2+*pTRC99a-*phoP* | This study |
| SL1344 ∆23+pPhoP | *∆sseK2/3+*pTRC99a-*phoP* | This study |
| plasmids |  |  |
| pET28a-*sseK1*(DXD) | endogenous promoter of *sseK1* | This study |
| pET28a-*sseK3*(DXD) | endogenous promoter of ss*eK3* | This study |
| pTrc99a-*phoP* | C-terminal with 1xFlag | This study |
| pTrc99a-*phoP*(R118A) | C-terminal with 1xFlag | This study |
| pET28a-*phoP* | PhoP expression plasmid | This study |
| pET28a-*phoP*(R65A) | PhoP mutant expression plasmid | This study |
| pET28a-*phoP*(R66A) | PhoP mutant expression plasmid | This study |
| pET28a-*phoP*(R118A) | PhoP mutant expression plasmid | This study |
| pET28a-*phoP*(R215A) | PhoP mutant expression plasmid | This study |
| pET28a-*phoP*(R65/66/118/215A) | PhoP mutant expression plasmid | This study |
| pGEX-*sseK1* | GST-SseK1 expression plasmid | This study |
| pGEX-*sseK3* | GST-SseK3 expression plasmid | This study |

**Table S2 Primers pairs for real-time PCR**

| **Primer name** | **Primer sequence (5’-3’)** |
| --- | --- |
| *sseK1* F | GGAAAACGGGATAATAGCTG |
| *sseK2* F | GAAAACATCACCAAACTGGA |
| *sseK3* F | TCAACAAAACCCCTATCTCA |
| *gyrB* F | CTGCTGTTGACCTTCTTCTAT |
| 16S F | GAAATGTTGGGTTAAGTCCC |
| *phoQ* F | TAATGCGCCGTAATAGCGGT |
| *phoP* F | CGAACGCCGTGAGTTTGATG |
| *pmrD* F | GGCGATATCCTGTCGCCTTT |
| *ssrB* F | AAAACCGTCGAAACACACCG |
| *mgtC* R | CAAGGGTTAGGTTCGGTCCC |
| *sseK1* R | TCAGAATAGGGATCAGCATC |
| *sseK2* R | TTTCCTCCGGATTGTTTTTC |
| *sseK3* R | TTGTCCAGCACTAAAATTCC |
| *gyrB* R | GTTCCTGCTTACCTTTCTTC |
| 16S R | CCTTCCTCCAGTTTATCACT |
| *phoQ* R | CGAACGCCGTGAGTTTGATG |
| *phoP* R | TAATGCGCCGTAATAGCGGT |
| *pmrD* R | GTCGCAGGAATAACAGCGTG |
| *ssrB* R | CCTCATTCTTCGGGCACAGT |
| *mgtC R* | TCAGCTCTCGCGTTTTACGA |
| *phoP* F-EcoRI | GCGCGAATTCATATGATGCGCGTACTGGTTGT |
| *phoP* F-XbaI | GGCCCCTCTAGATTAGCGCAATTCAAAAAGAT |
| R65A-F | CCTTAATAGCACGCTGGCGCAGCAGTGATGTTTCA |
| R65A-R | GCTGCGCCAGCGTGCTATTAAGGAAAGGCCGTCTT |
| R66A-F | CCTTAATACGCGCATGGCGCAGCAGTGATGTTTCA |
| R66A-R | GCTGCGCCATGCGCGTATTAAGGAAAGGCCGTCTT |
| R118A-F | AATGGCACGTAATAGCGGTCTGGCCTCCCA |
| R118A-R | ACCGCTATTACGTGCCATTAACGCCTGCAT |
| R215A-R | GCGCGCTCTAGATTAGCGCAATTCAAAAAGATATCCTTGTCCGGCTACGGTGGTAAT |
| *phoP* promoter-F | 6-FAM-TCGCGCTGTGACTCTGGTCG |
| *phoP* ptomoter R | 6-FAM-ATCCTCTACAACCAGTACGC |

**Table S3** **RNA-seq raw counts and SPI-2 gene expression**

**Table S4 Summary of Arg-GlcNAcylated peptides and proteins in mass spectrometry**

**Table S5 KEGG enrichment results**

|  | MainClass | Gene Hits In  Selected Set | All Genes In  Selected Set | Gene Hits  In Background | All Genes  In Background | p-value | Enrich Factor |
| --- | --- | --- | --- | --- | --- | --- | --- |
| 02020 Two-component system | Environmental Information Processing | 9 | 25 | 187 | 1666 | 0.000962 | 3.207273 |
| Signal transduction | Environmental Information Processing | 9 | 25 | 187 | 1666 | 0.000962 | 3.207273 |
| Environmental adaptation | Organismal Systems | 2 | 25 | 6 | 1666 | 0.003127 | 22.21333 |
| Organismal Systems | Organismal Systems | 2 | 25 | 7 | 1666 | 0.004337 | 19.04 |
| 01503 Cationic antimicrobial peptide (CAMP) resistance | Human Diseases | 3 | 25 | 37 | 1666 | 0.016571 | 5.403243 |
| Environmental Information Processing | Environmental Information Processing | 11 | 25 | 439 | 1666 | 0.041426 | 1.669795 |
| Genetic Information Processing | Genetic Information Processing | 6 | 25 | 197 | 1666 | 0.065399 | 2.029645 |
| Drug resistance | Human Diseases | 3 | 25 | 64 | 1666 | 0.068103 | 3.12375 |
| Translation | Genetic Information Processing | 3 | 25 | 81 | 1666 | 0.118111 | 2.468148 |
| 00310 Lysine degradation | Metabolism | 1 | 25 | 9 | 1666 | 0.127513 | 7.404444 |

**Table S6 GO enrichment results**

| GO_Term | S  gene number | TS  gene number | B  gene number | TB  gene number | p-value |
| --- | --- | --- | --- | --- | --- |
| phosphorelay signal transduction system | 6 | 36 | 40 | 3687 | 0.00 |
| translation elongation factor activity | 2 | 36 | 8 | 3687 | 0.00 |
| glycerol kinase activity | 1 | 36 | 1 | 3687 | 0.01 |
| dihydrolipoyllysine-residue succinyltransferase activity | 1 | 36 | 1 | 3687 | 0.01 |
| flavin adenine dinucleotide binding | 1 | 36 | 1 | 3687 | 0.01 |
| chemotaxis | 1 | 36 | 2 | 3687 | 0.02 |
| glycerol ether metabolic process | 1 | 36 | 2 | 3687 | 0.02 |
| cytosol | 1 | 36 | 5 | 3687 | 0.05 |
| phosphorelay response regulator activity | 1 | 36 | 5 | 3687 | 0.05 |
| RNA binding | 2 | 36 | 38 | 3687 | 0.05 |
| structural constituent of ribosome | 2 | 36 | 47 | 3687 | 0.08 |
| cytoplasm | 3 | 36 | 165 | 3687 | 0.22 |
| tRNA binding | 1 | 36 | 30 | 3687 | 0.26 |
| phosphorelay sensor kinase activity | 1 | 36 | 30 | 3687 | 0.26 |
| nucleic acid binding | 1 | 36 | 36 | 3687 | 0.30 |
| plasma membrane | 5 | 36 | 426 | 3687 | 0.40 |
| oxidoreductase activity | 1 | 36 | 57 | 3687 | 0.43 |
| DNA binding | 3 | 36 | 270 | 3687 | 0.50 |
| ATP binding | 1 | 36 | 183 | 3687 | 0.84 |
| integral component of membrane | 1 | 36 | 313 | 3687 | 0.96 |
